# Responsiveness to Influenza Vaccination Correlates with NKG2C-Expression on NK Cells

**DOI:** 10.3390/vaccines8020281

**Published:** 2020-06-05

**Authors:** Peggy Riese, Stephanie Trittel, Rishi D. Pathirana, Frank Klawonn, Rebecca J. Cox, Carlos A. Guzmán

**Affiliations:** 1 Department of Vaccinology and Applied Microbiology, Helmholtz Centre for Infection Research, 38124 Braunschweig, Germany; stephanie.trittel@helmholtz-hzi.de (S.T.); CarlosAlberto.Guzman@helmholtz-hzi.de (C.A.G.); 2 Department of Clinical Science, The Influenza Centre, University of Bergen, 5007 Bergen, Norway; rishi.Pathirana@gmail.com (R.D.P.); rebecca.cox@uib.no (R.J.C.); 3 K.G. Jebsen Centre for Influenza Vaccine Research, University of Oslo, 0313 Oslo, Norway; 4 Department of Biostatistics, Helmholtz Centre for Infection Research, 38124 Braunschweig, Germany; frank.klawonn@helmholtz-hzi.de; 5 Department of Computer Science, Ostfalia University of Applied Sciences, 38302 Wolfenbuettel, Germany; 6 Department of Research and Development, Haukeland University Hospital, 5021 Bergen, Norway; 7 Centre for Individualized Infection Medicine, 30625 Hannover, Germany

**Keywords:** influenza, vaccination, vaccine responsiveness, NK cells, NKG2C

## Abstract

Influenza vaccination often results in a large percentage of low responders, especially in high-risk groups. As a first line of defense, natural killer (NK) cells play a crucial role in the fight against infections. However, their implication with regard to vaccine responsiveness is insufficiently assessed. Therefore, this study aimed at the validation of essential NK cell features potentially associated with differential vaccine responsiveness with a special focus on NKG2C- and/or CD57-expressing NK cells considered to harbor memory-like functions. To this end, 16 healthy volunteers were vaccinated with an adjuvanted pandemic influenza vaccine. Vaccine responders and low responders were classified according to their hemagglutination inhibition antibody titers. A majority of responders displayed enhanced frequencies of NKG2C-expressing NK cells 7- or 14-days post-vaccination as compared to low responders, whereas the expression of CD57 was not differentially modulated. The NK cell cytotoxic potential was found to be confined to CD56^dim^CD16^+^ NKG2C-expressing NK cells in the responders but not in the low responders, which was further confirmed by stochastic neighbor embedding analysis. The presented study is the first of its kind that ascribes CD56^dim^CD16^+^ NKG2C-expressing NK cells a crucial role in biasing adaptive immune responses upon influenza vaccination and suggests NKG2C as a potential biomarker in predicting pandemic influenza vaccine responsiveness.

## 1. Introduction

Acute respiratory infections caused by the influenza virus are one of the major public health problems leading to high mortality rates worldwide, and consequently, to a large societal economic burden [1]. Occasionally, a novel virus arises, leading to worldwide spread and a pandemic. In the last decade, a number of pandemic influenza vaccines have received marketing authorization [2]. An increase in hemagglutination inhibition (HAI) antibody titer is commonly used to measure the response to the vaccine. However, antibody responses may be restricted in high-risk groups (i.e., the very young and the elderly, individuals with co-morbidities), resulting in a large percentage of low responders [3,4].

The generation of protective immunity requires the interplay between innate and adaptive immune cells. NK cells are described as crucial innate immune cells in the fight against influenza infections [5]. Recent findings in NK cell biology provide further evidence on specific functional features of NK cells, which highlight a formerly underestimated role during infection. Several studies revealed unexpected NK cell characteristics, like their continuous differentiation process and the impact of the education status on the magnitude of NK cell functionality [6,7,8,9]. Likewise, it was discovered that NK cells display adaptive immune features similar to T cells, including the generation of memory-like NK cells [10,11]. Several reports highlighted the occurrence of murine memory-like NK cells by (i) hapten-induced contact hypersensitivity, (ii) murine cytomegalovirus infection (MCMV) and (iii) cytokine stimulation [12,13,14,15]. Human memory-like NK cells were first described in vivo as a unique subset of NK cells expressing CD57 and NKG2C detected in a cohort of individuals infected with the human cytomegalovirus (HCMV) [16,17,18]. Subsequently, it was also demonstrated that human memory-like NK cells, characterized by the expression of NKG2C, could be induced in vitro by cytokine stimulation [19,20]. The CD94-NKG2C activating NK cell receptor binds to HLA-E and acts via the ITAM-bearing DAP12 signaling pathway [21]. CD57 is mainly expressed by terminally differentiated NK cells, which are suggested to have undergone clonal expansion following infection. CD57^+^ NK cells are described to harbor a lower proliferative capacity and to be less cytotoxic in response to cytokine stimulation, but show higher CD16-induced cytotoxicity [22].

Next to their newly discovered functional features, NK cells are well known to interact directly and indirectly with adaptive immune cells. Thus, it can be hypothesized that NK cells might be considered as relevant players in the initiation of adaptive immunity following influenza vaccination. Supporting evidence comes from a recent study, in which NK cell responsiveness following influenza vaccination was investigated. The results of this study demonstrated that NK cells with an intracellular immune memory, characterized by enhanced IFN*γ* secretion following antigen-specific re-stimulation, are generated following vaccination [23]. These NK cells displayed an increased internalization of the NKp46 receptor, which is known to interact with the influenza surface protein hemagglutinin (HA). However, despite this fragmentary evidence, there is still a considerable paucity of knowledge in this field. In this regard, NK cell subsets expressing CD57 and NKG2C have yet to be addressed. Thus, in the present study, the impact of the H1N1 vaccination on phenotypic and functional changes of NK cells expressing CD57 and NKG2C and their reciprocal influence on the vaccination efficacy was investigated.

## 2. Materials and Methods

### 2.1. Study Design

Sixteen healthy volunteers (health care workers (HCWs)) were vaccinated with the pandemic influenza vaccine Pandemrix^®^ (split virion, inactivated; A/California/07/2009 (H1N1)v-like strain (X-179A), GlaxoSmithKline, Brentford, UK), adjuvanted with AS03 as part of a large clinical trial. Fourteen of the participants were female and two were male (one normal and one low-responder), and they were born between 1951 and 1987 with a median birth year of 1974 and 1969 for normal- and low-responders, respectively. Other than three participants (normal responders), all participants received previous seasonal influenza vaccines. All participants provided written informed consent before inclusion in the study, which had ethical (Regional Committee for Medical Research Ethics (ethical approval number is 2009/1224, issued by REC west), Western Norway (REK Vest)) and regulatory (Norwegian Medicines Agency) approval and is registered at the National Institute for Health Database Clinical trials.gov (NCT01003288). Human subject rights were protected during the trial and the data analysis. Blood (clotted and Cell Preparation Tubes (CPTs)) was collected prior and 7-, 14-, 21- and 180-days post-vaccination [24]. Peripheral blood mononuclear cells (PBMCs) were isolated from CPT tubes according to the manufacturer’s instructions and cryo-preserved in 90% fetal bovine serum (FBS)/10% dimethyl sulfoxide (DMSO) until further analysis.

### 2.2. Humoral Immune Responses

The HAI titers in serum samples pre-vaccination and 7-, 14-, 21-, 90- and 180-days post-vaccination were determined by a HAI assay using the X179A virus. The assay was performed with 0.7% turkey red blood cells, as described previously [24]. The titers analyzed at days 0 and 90 were used to define responders and low responders. Vaccinees with a 4-fold seroconversion or a titer increase >40 were considered as responders. Human cytomegalovirus (CMV)-specific IgG antibodies were assessed using the Alinity i instrument (Abbott).

### 2.3. Cellular Immune Responses

PBMCs were thawed and 1 × 10^6^ to 4 × 10^6^ cells/sample were re-stimulated for 16 h in complete RPMI 1640 (Gibco, supplemented with 10% FCS, 5% Penicillin/Streptomycin and 5% Glutamine) containing the vaccine formulation with a final concentration of 4 µg hemagglutinin (HA)/mL split virus vaccine (kindly provided by GlaxoSmithKline, Belgium). Unstimulated samples were incubated for the same time in complete RPMI without the vaccine formulation. Brefeldin A and monensin were added to all samples after 5 h of incubation. Cells were collected and stained for flow cytometric analysis. Surface marker staining was performed for 20 min at 4 °C. The following antibodies were used diluted in PBS: CD56 (PE-Cy7, clone B159, BD, Franklin Lakes, NJ, USA), CD3 (V450, clone UCHT1, BD), CD14 (Pacific Blue, clone M5E2, BD), CD19 (V450, clone HIB19, BD Horizon), CD16 (APC-H7, clone 3G8, BD Pharmingen), NKG2C (PE, clone 134591, R&D Systems, Minneapolis, MN, USA), CD57 (APC, clone HCD57, BioLegend, San Diego, CA, USA), Live/Dead (Fixable Blue, Invitrogen, Carlsbad, CA, USA). The expression of CD107a was used as a correlate of degranulation. To this end, the anti-CD107a antibody (PE-Cy5, clone eBioH4A3, eBioscience, San Diego, CA, USA) was added to the culture. The secretion of IFN*γ* (Alexa Fluor 700, clone B27, BioLegend) was detected by intracellular staining using Cytofix/Cytoperm solution (BD Biosciences). Samples were acquired at a BD Fortessa flow cytometer and analyzed using FlowJo (FlowJo, LLC, Ashland, OR, USA). Unstained, single stained (one antibody/sample) as well as fluorescence-minus-one (FMO) samples were used as controls for the acquisition as well as the subsequent analysis. Statistical differences were determined by the GraphPad Prism software.

### 2.4. Stochastic Neighbor Embedding (SNE) Analysis

Flow cytometry data of responders and low responders derived pre- and 7-days post-vaccination were imported into FlowJo (version 9) and compensation channel values were extracted for the following parameters: CD56, CD16, NKG2C, CD57, CD107a and IFN*γ*. Up to 10,000 values were extracted per vaccinee and time point and then pooled for responders and low responders. By using the R package “tsne”, a t-distributed SNE analysis using Barnes–Hut implementation was performed and the resulting data were plotted with intensities for the depicted markers (RStudio version 3.2.1, RStudio, Inc., Boston, MA, USA), as described earlier [25,26].

### 2.5. Statistical Analysis

GraphPad Prism (version 6.0 for Windows, GraphPad Software, La Jolla, CA, USA) was used for the statistical assessment (unpaired low-parametric Mann–Whitney or Kruskal–Wallis test and Spearman correlation). Values of *p* ≤ 0.05 were considered significant.

## 3. Results

### 3.1. Influenza Vaccination Leads to Enhanced Frequencies of NKG2C-Expressing NK Cells

**Figure 1:**
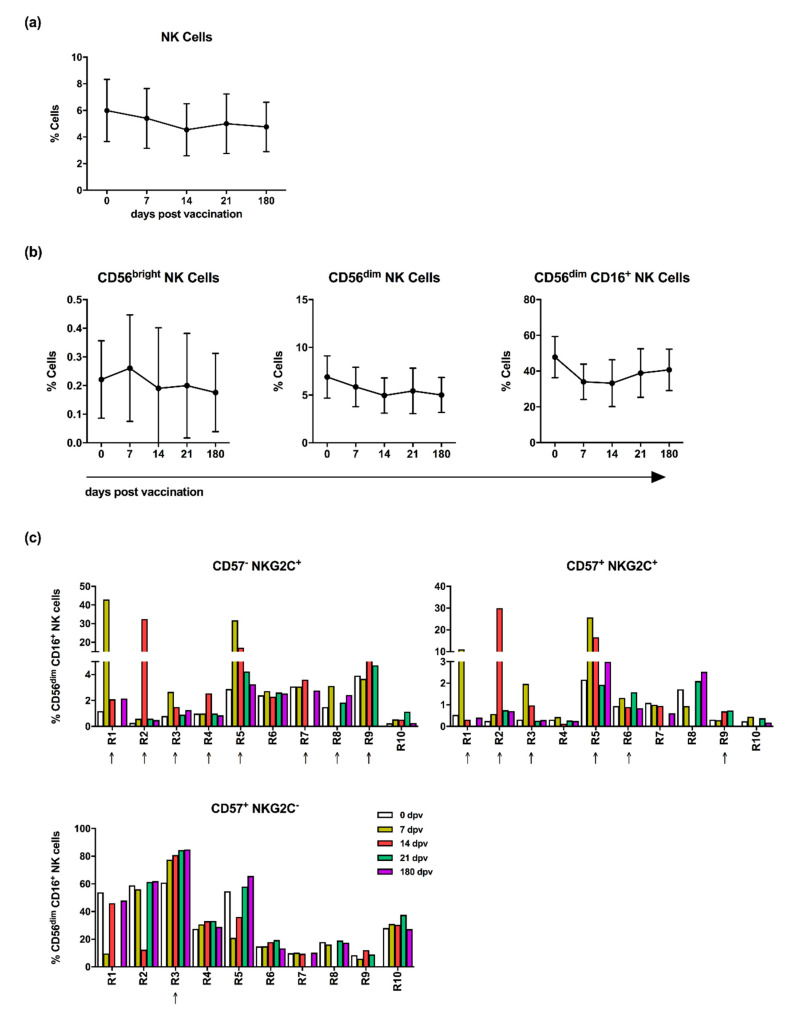

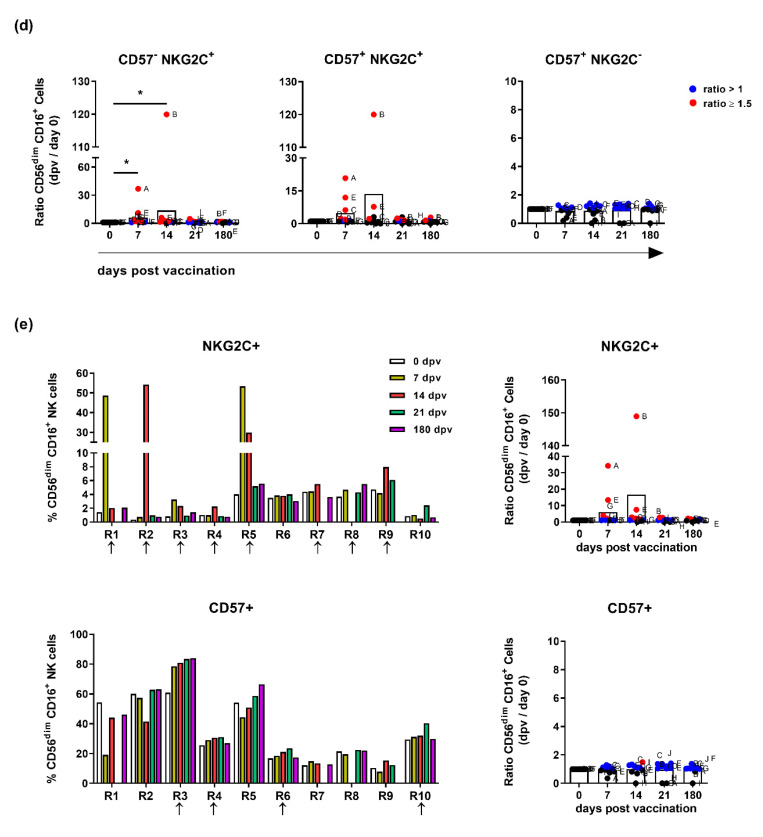
Influenza vaccination affects the frequency of NKG2C-expressing natural killer (NK) cells. Peripheral blood mononuclear cells (PBMCs) isolated from vaccinated individuals prior to vaccination and at the indicated time points post-vaccination (dpv = days post-vaccination) were stained for the surface markers CD56, CD3, CD16, NKG2C and CD57. (**a**) Frequencies of total CD3^−^CD56^+^ NK cells and (**b**) of CD3^−^CD56b^right^, CD3^−^CD56^dim^ and CD3^−^CD56^dim^CD16^+^ NK cell subpopulations. Diagrams show the connected column mean with 95% confidence interval. (**c**) Frequencies of NK cell populations characterized by the expression of CD57 and NKG2C. Columns represent individual data points. (**d**) CD57- and NKG2C-expressing CD56^dim^CD16^+^ subpopulations depicted as the ratio of cell frequencies detected at the indicated time points post-vaccination and the frequencies detected prior vaccination (day 0). Diagrams are depicted as scatter plots with bars of individual assigned data points. (**e**) CD56^dim^CD16^+^NKG2C^+^ and CD56^dim^CD16^+^CD57^+^ NK cells displayed as frequencies and as the ratio to the day before vaccination. Columns represent individual data points; diagrams are depicted as scatter plots with bars of individual assigned data points. Arrows indicate donors with vaccine-induced immunological changes. Asterisks denote significant values as calculated by unpaired and non-parametric Kruskal–Wallis test. * *p* ≤ 0.05. Letters in panels (**d**, **e**) indicate single responders.

NK cells are characterized by the intensity of CD56 expression and the co-expression of CD16 and can be thereby divided into different functional subsets (see gating strategy, Figure S1). The impact of pandemic vaccination on the distribution of blood NK cell subsets was assessed by flow cytometry at various time points. A slightly reduced frequency of total NK cells in vaccinated individuals was observed during the first 14 days after vaccination (≈6% at day 0 to ≈5.4% at day 7 and 4.5% at day 14) (Figure 1a). The marginally diminished frequency remained relatively stable over the observation period until day 180 post-vaccination (≈5% at day 21 and ≈4.8% at day 180). The division of NK cells into primarily cytokine secreting CD56^bright^ and highly cytotoxic CD56^dim^ subsets revealed a decrease in the CD56^dim^ NK cell frequencies, especially of CD56^dim^CD16^+^ cells (≈48% at day 0 to ≈34% at day 7), whereas CD56^bright^ NK cell frequencies were not affected (Figure 1b). These data suggest that vaccination-induced modulation mainly affects CD56^dim^CD16^+^ NK cells, which are described to hold a higher cytotoxic but lower cytokine secreting capability as compared to CD56^bright^ NK cells [27]. Human memory-like NK cells based either on the expression of CD57 and NKG2C or NKG2C alone were recently described to exert amplified recall responses upon CMV infection or to be induced after cytokine stimulation (IL-12, IL-15 and IL-18), respectively [13,17,20]. The analysis of CD56^dim^CD16^+^ NK cells with regard to CD57 and NKG2C expression after pandemic vaccination revealed that the changes in NK cell frequencies were mainly restricted to the NKG2C-expressing subsets. Increased frequencies of both CD57^−^NKG2C^+^ (from ≈2% at day 0–9% at day 7 and ≈8% at day 14) and CD57^+^NKG2C^+^ NK cell subsets (from ≈1% at day 0–4% at day 7 and ≈7% at day 14) were detected at days 7 and 14 post-vaccination (orange and red) in samples derived from 8 out of 10 vaccine responders (marked with an arrow, Figure 1c). The most striking differences were observed at day 7 and 14 post-vaccination, with significantly elevated levels of both CD57^−^NKG2C^+^ and CD57^+^NKG2C^+^ NK cells, whereas the frequency of CD57^+^NKG2C^−^ NK cells was not affected (Figure 1d). Within the NKG2C-expressing subsets, high ratios comparing post- and pre-vaccination values were detected (≥1.5, red data points) while the CD57^+^NKG2C^−^ NK cell subset showed ratios > 1 (blue data points), but not ≥1.5. The analysis of NK cells either expressing NKG2C or CD57 confirmed the increased frequency of NKG2C but not CD57 expression (Figure 1e). The data suggest that adjuvanted pandemic vaccination induces changes in the subset of NKG2C-expressing NK cells, which in turn can be potentially involved in determining the outcome of vaccination. While the frequency of NKG2C-expressing NK cells is described to be connected with CMV sero-positivity, no such correlation was found here (Figure S2a) [16,17]. Furthermore, no differences between the mean CMV titer of low and normal responders was observed (Figure S2b). The correlation analysis of the CMV titer and the fold change of the HAI titer or the age of the participants also did not yield any significant relation (Figure S2c). Likewise, the fold change of the HAI titer did not correlate with the age of the vaccinees (Figure S2d). However, in normal but not in low responders, the frequency of NKG2C^+^CD57^+^ NK cells at day 7 post-vaccination showed a significant negative correlation with the age of the vaccinees (Figure S2e). These findings highlight that the hypothesized relation between NKG2C^+^ NK cells and the vaccination outcome is not dependent on the CMV sero-status, thus supporting publications stating that CMV infections do not affect influenza vaccine efficacy [28].

### 3.2. NK Cells of Influenza Vaccination Responders and Low Responders Display Differences in NKG2C and CD57 Expression

**Figure 2:**
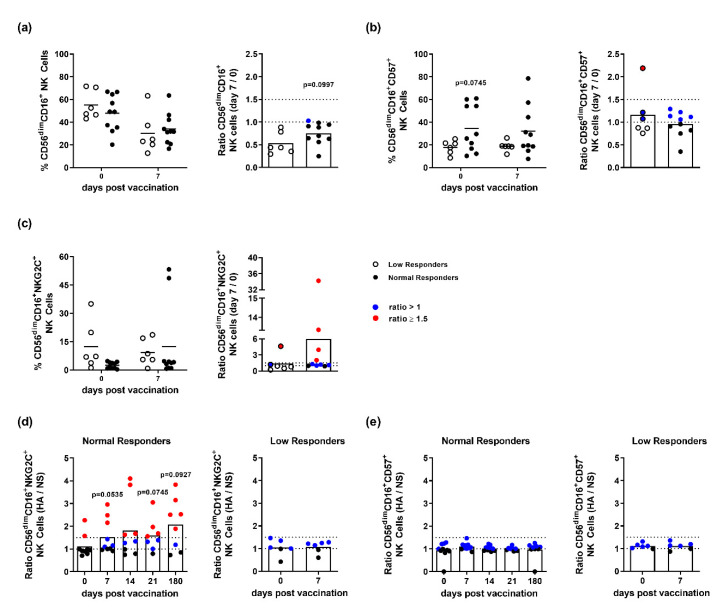
Phenotypic analysis of NK cells derived from normal and low responders to the pandemic influenza vaccination. Flow cytometric analysis of frozen PBMCs isolated from vaccinated individuals classified into normal responders (black dots) and low responders (white dots; a-c). Frequencies and ratios of (**a**) CD56^dim^CD16^+^, (**b**) CD56^dim^CD16^+^CD57^+^ and (**c**) CD56^dim^CD16^+^NKG2C^+^ NK cells. Diagrams are depicted as scatter dot plots indicating the mean by a horizontal line. Ratios were derived from cell frequencies detected at day 7 post-vaccination and the frequencies detected prior to vaccination (day 0) depicted as scatter plots with bars. Ratios of unstimulated (NS) and HA-re-stimulated (HA) (**d**) CD56^dim^CD16^+^NKG2C^+^ and (**e**) CD56^dim^CD16^+^CD57^+^ NK cells derived from normal and low responders. Blue dots depict ratios >1 and red dots depict values ≥ 1.5.

The inefficacy of influenza vaccines, characterized by the varying occurrence of low responders, is a persisting problem. To classify normal and low responders following pandemic vaccination, the HAI titer of each vaccinee was evaluated at days 0 and 90 post-vaccination (Figure S3). The total frequencies of NK cells derived from normal and low responders were compared, as well as the expression of CD57 and NKG2C. Reduced frequencies of CD56^dim^CD16^+^ NK cells were observed in both response groups at 7-days post-vaccination, as compared to day 0. However, the decrease was less profound in normal responders (≈30% reduction for responders and ≈46% reduction for low responders) (Figure 2a). These findings were consistent with the assessed ratios of NK cell frequencies (day 7/day 0). The assessment of CD57 expression prior to vaccination demonstrated differences in its basal expression by NK cells derived from normal and low responders (day 0). However, the ratio of CD56^dim^CD16^+^CD57^+^ NK cell frequencies (day 7/day 0) revealed no vaccine-induced effect on CD57 expression (ratios ≈1 for both responders and low responders (Figure 2b). The analysis of CD56^dim^CD16^+^NKG2C^+^ NK cells revealed an increased frequency at day 7 post-vaccination in normal responders that was not observed in low responders (Figure 2c). The ratio of CD56^dim^CD16^+^NKG2C^+^ NK cell frequencies (day 7/day 0) supports this finding by displaying a higher ratio for normal responders (≈6) as compared to low responders (≈1). This difference is further highlighted by ratios mainly ≥1.5 (red dots) detected for NKG2C-expression by CD16^+^ NK cells derived from normal responders. This indicates that responders show increased frequencies of NKG2C^+^ expression at day 7 post-vaccination as compared to day 0, whereas NKG2C expression in low responders remains largely unaffected. To address whether the observed increased frequency of NKG2C^+^ NK cells is due to the specific antigen (HA) re-stimulation, an individual analysis of single donors was performed. These data revealed that a significant number of responders displayed an HA-induced surface expression of NKG2C that was not observed in the group of low responders (Figure 2d, ratio (HA/unstimulated (NS)) >1 = blue, ≥1.5 = red). Interestingly, the individuals responding to HA re-stimulation with enhanced expression of NKG2C already displayed a higher basal expression. With regard to the HA-induced surface expression of CD57, neither vaccine responders nor low responders displayed a strong modulation, as indicated by ratios (HA/unstimulated) >1 but not ≥1.5 (Figure 2e). These findings suggest that NKG2C-expressing NK cells bias vaccine responsiveness and might serve as a determinant of responsiveness towards influenza vaccination.

### 3.3. CD107a Expression Is Confined to CD56^dim^CD16^+^NKG2C-Expressing NK Cells in Responders but Not in Low Responders

**Figure 3:**
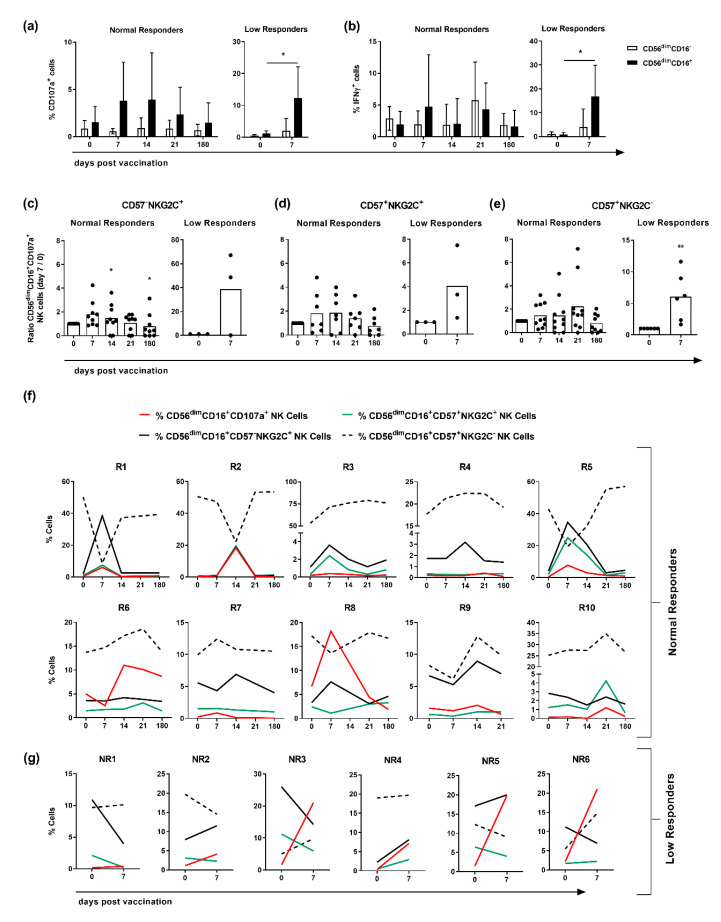
Functional differences characterize NK cells isolated from normal and low responders. Functional flow cytometric analysis of frozen PBMCs isolated from vaccinated individuals classified into normal and low responders. Frequencies of (**a**) CD107a^+^CD56^dim^ and (**b**) IFNγ^+^CD56^dim^ NK cells with regard to CD16 expression (CD16^−^ white/CD16^+^ black). Ratio of CD107a-expressing (**c**) CD57^−^NKG2C^+^, (**d**) CD57^+^NKG2C^+^ and (**e**) CD57^+^NKG2C^−^ CD56^dim^CD16^+^ NK cells isolated from responders and non-responders on the indicated days post- and pre-vaccination (day 0) depicted as scatter plots with bars indicating the mean with 95% confidence interval. (**f**) Frequency of CD56^dim^CD16^+^ NK cells expressing CD107a (red solid), NKG2C^+^CD57^−^ (black solid), NKG2C^+^CD57^+^ (green), NKG2C^−^CD57^+^ (black dashed) shown for individual responders and (**g**) non-responders. Asterisks denote significant values as calculated by unpaired and non-parametric Mann–Whitney test. * *p* ≤ 0.05, ** *p* ≤ 0.01.

To dissect whether, in addition to the phenotypic alterations, the functionality of CD56^dim^ NK cells in normal and low responders also differs, CD16 expression, CD107a expression and IFN*γ* secretion were addressed ex vivo. Independently of the responsiveness to vaccination, differences in the functionality of CD16^−^ and CD16^+^ NK cells within the CD56^dim^ subset were detected (Figure 3a,b). The analysis of the NK cell degranulation capacity revealed that within the group of responders, CD56^dim^CD16^−^ NK cells showed no changes in the frequency of CD107a-expressing cells after vaccination (Figure 3a). In contrast, CD56^dim^CD16^+^ NK cells derived from responders exhibited an enhanced expression of CD107a peaking at day 7 post-vaccination. Low responders showed similar functional differences between CD56^dim^CD16^−^ and CD16^+^ NK cells (Figure 3a). CD56^dim^CD16^+^ NK cells showed a higher frequency of CD107a-expressing cells at day 7 post-vaccination as compared to day 0. The comparison of normal and low responders further revealed that responders harbor a lower frequency of CD56^dim^CD16^+^CD107a^+^ NK cells at day 7 post-vaccination (≈4%) as compared to low responders (≈12%) (Figure 3a).

The analysis of IFN*γ*-expressing CD56^dim^ NK cell subsets derived from responders revealed that vaccination differentially impacts CD16^−^ and CD16^+^ NK cells only at day 7 post-vaccination as compared to all other investigated time points (day 0, 14, 21 and 180 post-vaccination) (Figure 3b). Normal responders harbored a lower frequency of CD56^dim^CD16^+^IFN*γ*^+^ NK cells at day 7 post-vaccination (≈5%) as compared to low responders (≈17%) (Figure 3b).

An investigation of the NK cell functionality depending on CD57 and NKG2C expression revealed elevated levels in the expression of CD107a in normal and low responders. Interestingly, within the subset of CD57^−^NKG2C^+^ NK cells, low responders showed a 22-fold higher CD107a expression as compared to responders at day 7 (Figure 3c). Within the subset of CD57^+^NKG2C^+^ NK cells, the comparison of normal and low responders showed less profound differences (3.5 fold increase) (Figure 3d). CD107a expression within the CD57^+^NKG2C^−^ NK cell subset showed marginal changes in responders (≈1.5-fold), whereas low responders displayed a six-fold increase at day 7 post-vaccination (Figure 3e).

The analysis of individual responders highlights distinct, donor specific differences within the groups of normal and low responders. Thus, five out of 10 responders (R1, R2, R5, R8, and R9) showed enhanced frequencies of both NKG2C-expressing NK cell subsets at day 7 or 14 after vaccination as compared to day 0 (Figure 3f). This increase appears to be associated with an elevated level of CD107a as well as a decreased frequency of CD57^+^NKG2C^−^ NK cells. Interestingly, the CD57^+^NKG2C^+^ subset seems to be strongly associated with the expression of CD107a as compared to the CD57-NKG2C+ subset in six out of 10 normal responders (Figure 3f). On the other hand, five out of six low responders (NR1, NR2, NR3, NR5, and NR6) did not show this correlation. These results suggest that the functionality of NK cells derived from responders is confined within both NKG2C-expressing subpopulations.

### 3.4. Elevated NKG2C-Expression Correlates with a Higher Expression of CD107a

**Figure 4:**
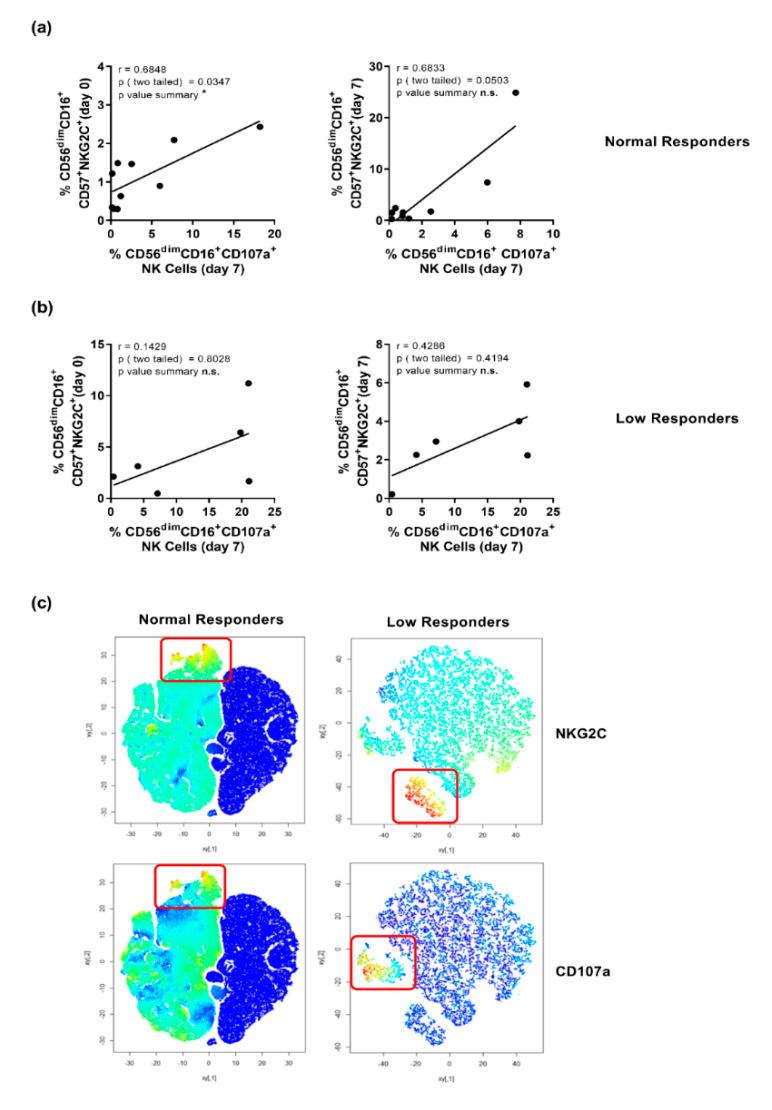
Responders display a correlation of NKG2C with CD107a-expressing NK cells. Flow cytometry derived data from frozen PBMCs isolated from vaccinated individuals classified into responders and non-responders. Correlation of CD57^+^NKG2C^+^CD56^dim^CD16^+^ NK cell frequencies detected prior to vaccination (day 0) or at day 7 post-vaccination with the frequency of CD107a^+^CD56^dim^CD16^+^ NK cells detected at day 7 post-vaccination depicted for (**a**) responders and (**b**) non-responders. The black line depicts the linear regression; calculations were performed by Spearman correlation analysis. Asterisks indicate significant correlations according to * *p* ≤ 0.05; n.s. = not significant. (**c**) T-distributed SNE (*t*SNE) analysis of flow cytometry data derived from normal and low responders before and at day 7 post-vaccination. Cells displaying a high (**red**) or low (**blue**) expression density of NKG2C (upper panel) and CD107a (lower panel) are depicted for responders (**left**) and non-responders (**right**).

A correlation analysis of the frequency of CD56^dim^CD16^+^CD57^+^NKG2C^+^ NK cells on day 0/7 post-vaccination and the degranulation capacity of CD56^dim^CD16^+^ NK cells (frequency of CD107a^+^ NK cells) on day 7 revealed a substantial positive correlation for NK cells derived from normal responders (Figure 4a). Within the group of low responders, no significant correlation between the frequency of CD56^dim^CD16^+^CD57^+^NKG2C^+^ NK cells and their CD107a expression at days 0 or 7 post-vaccination was found (Figure 4b). An SNE analysis confirmed that CD56^dim^CD16^+^ NK cells derived from normal responders prior and 7-days post-vaccination overlap with regard to the expression of NKG2C and CD107a as detected by flow cytometry. Interestingly, this overlap was not detected for low responders (Figure 4c). The obtained data suggest that CD107a expression within the CD56^dim^CD16^+^NKG2C^+^ NK cell subset might represent an early correlate of vaccine responsiveness.

## 4. Discussion

NK cells represent an innate immune cell population that acts as a first line of defense by killing viral infected cells and regulating cells of the adaptive immune system by means of secreted cytokines. This renders NK cells as promising immune players to be considered for the development of vaccination strategies against viral pathogens. The few studies investigating NK cells following the vaccination of healthy adults revealed their beneficial effect against a variety of pathogens. Enhanced frequencies of IFNγ-secreting NK cells upon malaria vaccination were associated with antigen-specific IL-2 secretion and considered as a marker for antigen-specific T cell activation [29]. Vaccination against the rabies virus demonstrated a significant impact of NK cell activation for vaccine-induced effector as well as recall responses [30]. Furthermore, reduced viral loads upon vaccination with live attenuated yellow fever vaccine correlated with enhanced NK cell functionality, such as activation and degranulation [31]. Influenza vaccines were reported to induce changes in NK cell receptor repertoires and to promote increased IFNγ secretion [32]. A reduced CD16 expression by NK cells was also described in the context of a robust influenza-specific IgG response [33]. However, there is a paucity of knowledge concerning the potential implications of NK cells in responsiveness to influenza vaccines. To the best of our knowledge, this is the first study aiming at the validation of phenotypic and functional differences of NK cells isolated from vaccine responders and low responders.

The analysis of peripheral blood NK cell frequencies upon immunization with the adjuvanted pandemic H1N1 vaccine revealed a slight decrease over time. The observed decrease might result from activation and subsequent migration to secondary lymphoid organs. Interestingly, at day 7 post-vaccination, increased frequencies of CD56^dim^CD16^+^NKG2C^+^ NK cells were detected within the group of vaccine responders as compared to low responders. The increased expression of NKG2C upon influenza vaccination was observed regardless of CD57 expression. NKG2C-expressing NK cells were demonstrated to respond in vitro to cytokine stimulation [20]. Beneficial implications for NKG2C^+^ NK cells in natural infections with HIV, HBV, Hanta and Chikungunya viruses were recently shown by other groups [34,35,36]. However, few studies addressed the effect of vaccination on NKG2C-expressing NK cells. The vaccination of healthy volunteers with a low-adjuvanted split influenza vaccine or with a low-adjuvanted dendritic cell-based HIV vaccine revealed no major changes in the frequency of NKG2C- and/or CD57-expressing NK cells [23,37]. These findings might suggest that the observed increase in NKG2C-expressing NK cells upon pandemic influenza vaccination is due to adjuvantation with AS03. However, the immunization of macaques with a live attenuated SIV vaccine and a subsequent challenge was found to result in elevated frequencies of NKG2C-expressing circulating T cells in vaccinated but not unvaccinated animals [38]. Another study with SIV-infected rhesus macaques demonstrated the NK cell-mediated killing of antigen-pulsed DCs in a NKG2C-dependent manner [39].

The gain of CD57 expression is associated with immune cell maturation and senescence [21,40,41]. In the present study, responders displayed a higher basal level of CD57^+^ NK cells compared to low responders. However, the influenza vaccination itself did not result in any further changes. The functional analysis of CD56^dim^CD16^+^ NK cells at day 7 post-vaccination pointed to both responders and low < responders displaying enhanced IFN*γ* secretion and CD107a expression. This is in agreement with a previous study showing increased IFN*γ*-secretion following seasonal influenza vaccination [32]. The present work revealed that NK cells derived from responders secrete less IFN*γ* and show a reduced degree of degranulation as compared to low responders. Recent reports describe that NK cells activated by immunization or viral infections can lyse activated T cells, and thus prevent the efficient formation of a memory T cell pool required for long-term protective immunity [42,43,44]. This might explain, at least in part, the reduced humoral immune response defining low responders. Therefore, an enhanced NK cell functionality might result in a decreased memory T cell pool, and thus a reduced vaccine efficacy.

Taking into consideration the expression of CD57 and NKG2C, the analysis of the degranulation capacity revealed a correlation between CD107a expression in CD57^−^NKG2C^+^ and CD57^+^NKG2C^+^ NK cell populations and the overall responsiveness to vaccination. Recent studies described the generation of highly degranulating and IFN*γ*-secreting NKG2C^+^ NK cells upon CD16 cross-linking or by the activation of natural cytotoxicity receptors [36,45]. Furthermore, CD57^+^ NK cells are reported to exhibit a high basal cytotoxic potential [41]. Thus, the observed high basal level of CD57^+^ NK cells in responders might contribute to influenza vaccine responsiveness. In this regard, the significant correlation of CD56^dim^CD16^+^CD57^+^NKG2C^+^ frequencies pre-vaccination with the NK cell degranulation capacity found in responders but not for low responders suggests that the NK cell status at basal level and early upon vaccination might contribute to the overall vaccine responsiveness. The *t*SNE analysis confirmed the positive correlation and a two-dimensional overlap of degranulation and NKG2C-expressing NK cells that was not observed for low responders. In murine studies, degranulating NK cells were described to harbor immune-regulatory functions as well as to imprint adaptive immune responses [42]. Thus, NK cell-mediated antiviral activity via cytotoxicity was shown to promote early CD8 T cell responses [43]. Furthermore, NK cells can contribute to improved antigen-presentation by killing infected cells, thereby resulting in antigen-specific CD4 T helper cell responses and subsequent stimulation of humoral immunity [46]. Therefore, CD107a^+^NKG2C-expressing NK cells might bias vaccine responsiveness. A seasonal influenza vaccine study revealed enhanced frequencies of CD107a NK cells induced by increased IL-2 levels and immune complexes [47]. Interestingly, in the present study, responders displayed enhanced numbers of IL-2 secreting T cells as compared to low responders. Thus, the increased amount of IL-2 might affect the NK cell degranulating capacity (data not shown).

Although, CD56^dim^CD16^+^ NK cells displayed differences in the level of IFNγ secretion in responders and low responders, no correlation to either CD57 or NKG2C expression was detected (data not shown). A similar functional dichotomy of NKG2C^+^ NK cells was observed in patients suffering from chronic HCV infection in which an altered phenotype defined by enhanced frequencies of NKG2C^+^ NK cells was associated with enhanced expression of CD107a but not increased secretion of IFN*γ* [48]. Thus, a differential activation of degranulating or cytokine-secreting NKG2C^+^ NK cells might be similarly induced by the adjuvanted influenza vaccine.

Interestingly, human NKG2C-expressing NK cells are described to show memory-like features (e.g., clonal expansion) and enhanced specific recall responses such as proliferation and cytokine secretion [13,18,49,50]. Thus, the observed increased expression of NKG2C in responders might suggest a vaccine-induced generation of an NK cell pool with memory-like features. A previous influenza infection or immunization might have contributed to the formation of antigen-specific NKG2C-expressing NK cells, which can rapidly be activated after vaccination. The increased expression of NKG2C at days 7 and 14 post-vaccination in a few responders following HA re-stimulation supports this hypothesis. In a murine model, the generation of influenza-specific memory-like NK cells characterized by the expression of CXCR6 was reported [12]. It is known that the human receptor NKp46 binds specifically to influenza HA on the surface of infected cells [51]. Recent studies described NKp46-internalization as a distinct feature of influenza-specific memory-like NK cells [23]. In line with the concept of memory-like NK cells, antigen-specific recall responses were also reported for NK cells isolated from SIV-infected macaques, as well as in cells from human pleural fluid derived from tuberculosis patients upon re-stimulation with the Bacillus Calmette-Guérin vaccine [39,52]. These findings support a crucial role of CD56^dim^CD16^+^NKG2C^+^ NK cells for determining the outcome of vaccination. Interestingly, the phenotype of memory-like NK cell populations in humans is not consistent in the different models investigated to date. For example, CMV-induced memory-like NK cells express both NKG2C and CD57, whereas in vitro cytokine-induced memory-like NK cells are defined by the expression of NKG2C, amongst other markers, but the lack of CD57 expression [16,19,20]. The enhanced basal frequencies of CD57^+^NKG2C^+^-expressing NK cells in CMV-positive individuals [53] might raise concerns about whether our findings are due to an underlying chronic CMV infection. However, the increased expression of NKG2C at days 7 and 14 ex vivo and upon HA re-stimulation strongly argues for an influenza vaccination-induced effect. With regard to demographic information of the participants, no correlation explaining a vaccine bias could be detected. Furthermore, it should be noted that a small number of participants was involved in the presented study arm (*n* = 16). Additional, more extensive studies will be needed to confirm the presented data and to consider including NK cell targeting in future vaccination strategies.

The differentiation status of CD56^dim^CD16^+^ NK cells, defined by the expression of NKG2A, KIR and CD57, dictates their functionality [7,8]. The efficacy of the yellow fever vaccination was associated with a less differentiated NK cell phenotype defined by lack of CD57. However, it seems that vaccination with an adjuvanted pandemic vaccine does not affect NK cell differentiation at the analyzed time points (data not shown). This is consistent with another study, which investigated the effect of a seasonal influenza vaccination on NK cell differentiation and functionality [32].

Natural killer T (NKT) cells are described to bridge the innate and adaptive immune system by expressing surface markers characteristic for NK as well as for T cells, e.g., CD3 and CD56. Although in the murine system they were described to improve influenza-specific innate as well as adaptive immune responses, their impact in the human system is insufficiently studied [54,55]. Activation of NKT cells by the lipid antigen *α*-galactosylceramide or its analogs is discussed in the context of novel vaccination strategies [56,57]. They are further known to directly impact NK cell functionality via the secretion of activating (e.g., IFN*γ* and TNF*α*) as well as inhibitory cytokines (e.g., IL-4 and IL-10). Therefore, assessing the specific impact of NKT cells in the course of influenza infection or vaccination in future studies in the human system will help to further elucidate potential innate immune mechanisms affecting vaccine responsiveness.

The vaccine formulation used in this study contains the oil-in-water emulsion-based adjuvant system 03 (AS03). In accordance with earlier reports, the adjuvant contributes to enhanced antigen loading and presentation by antigen-presenting cells (APCs) and the generation of chemokine and cytokine gradients recruiting inflammatory cells to the site of injection [58,59]. NK cells are directly activated by binding of influenza-induced ligands, e.g., on infected DCs, to NKG2D and NKp46 (recognizing the viral HA) [60]. These interactions, as well as HA-recognition by co-stimulating molecules, such as 2B4 and NTB-A, lead to the elimination of infected cells by NK cell cytotoxicity [61]. Furthermore, the binding of CD16, expressed by NK cells, to the Fc region of surface-bound antibodies against viral proteins leads to the activation of antibody-dependent cellular cytotoxicity mediated by NK cell-derived granzyme B and perforin [62]. The AS03-enhanced antigen-presentation and cytokine secretion, e.g., by DCs, macrophages, monocytes, and neutrophils might thereby indirectly affect the phenotype of human NK cells and correspondingly increase their functionality. Nevertheless, regardless of the specific formulation, the data presented here clearly demonstrated the impact of vaccination on NK cell population composition and functionality, as well as the fact that changes in NK cell phenotype and functionally correlate with vaccine responsiveness. Therefore, acquisition of NKG2C might represent one potential early determinant to differentiate between responders and low responders, as well as be a driving force to promote efficacious adaptive responses post-vaccination.

## Supplementary Materials

The following are available online at https://www.mdpi.com/2076-393X/8/2/281/s1, Figure S1: Gating Strategy; Figure S2: Correlation analyses; Figure S3: Classification of vaccine responders and non-responders.
