# Supplementary material for: Responsiveness to Influenza Vaccination Correlates with NKG2C-Expression on NK Cells
Source: Vaccines (Basel). 2020 Jun 5;8(2):281. doi: 10.3390/vaccines8020281 (PMC7349951; doi:10.3390/vaccines8020281)
Supplement: Supplementary file 1 [file vaccines-08-00281-s001.pdf]

Supplementary Materials:

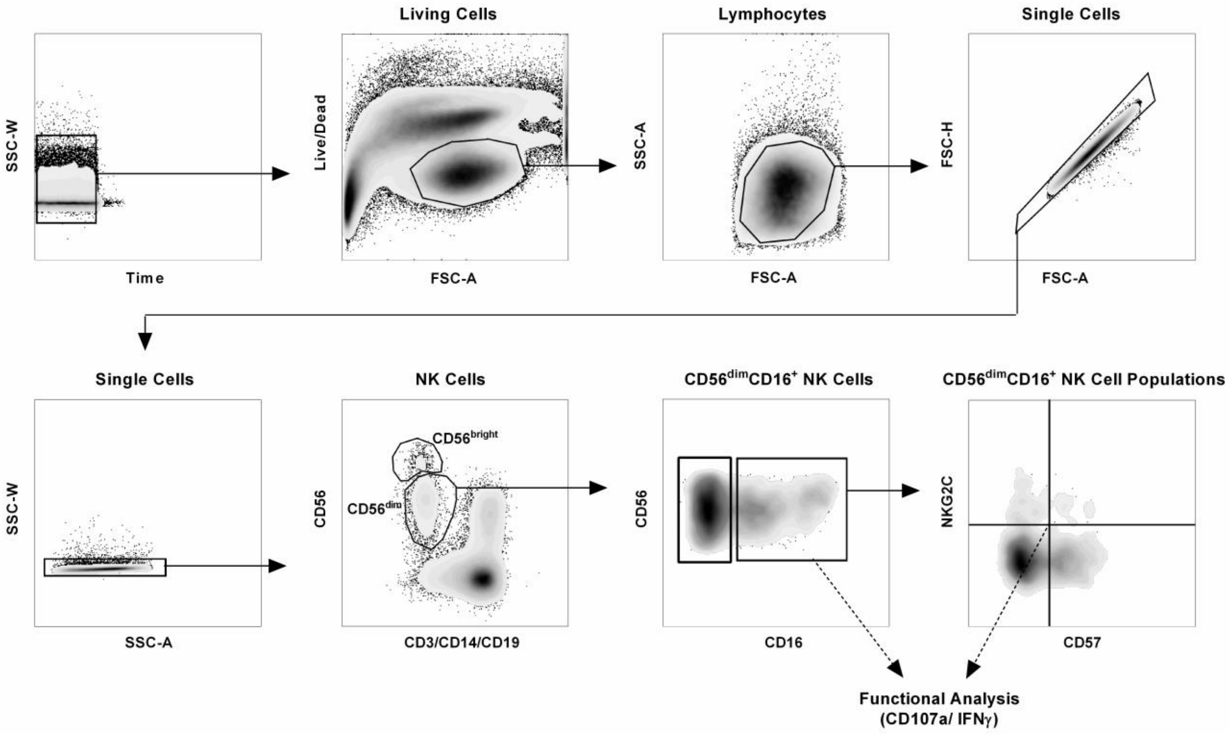

Figure S1. Gating Strategy. Measurement inconsistencies were excluded by using a time gate. Living single cell lymphocytes were gated on lineage negative (CD3/CD14/CD19) and CD56^{bright} and CD56^{dim} NK cells that were further characterized by the expression of CD16, CD57 and NKG2C and functionally analyzed.

Figure S2

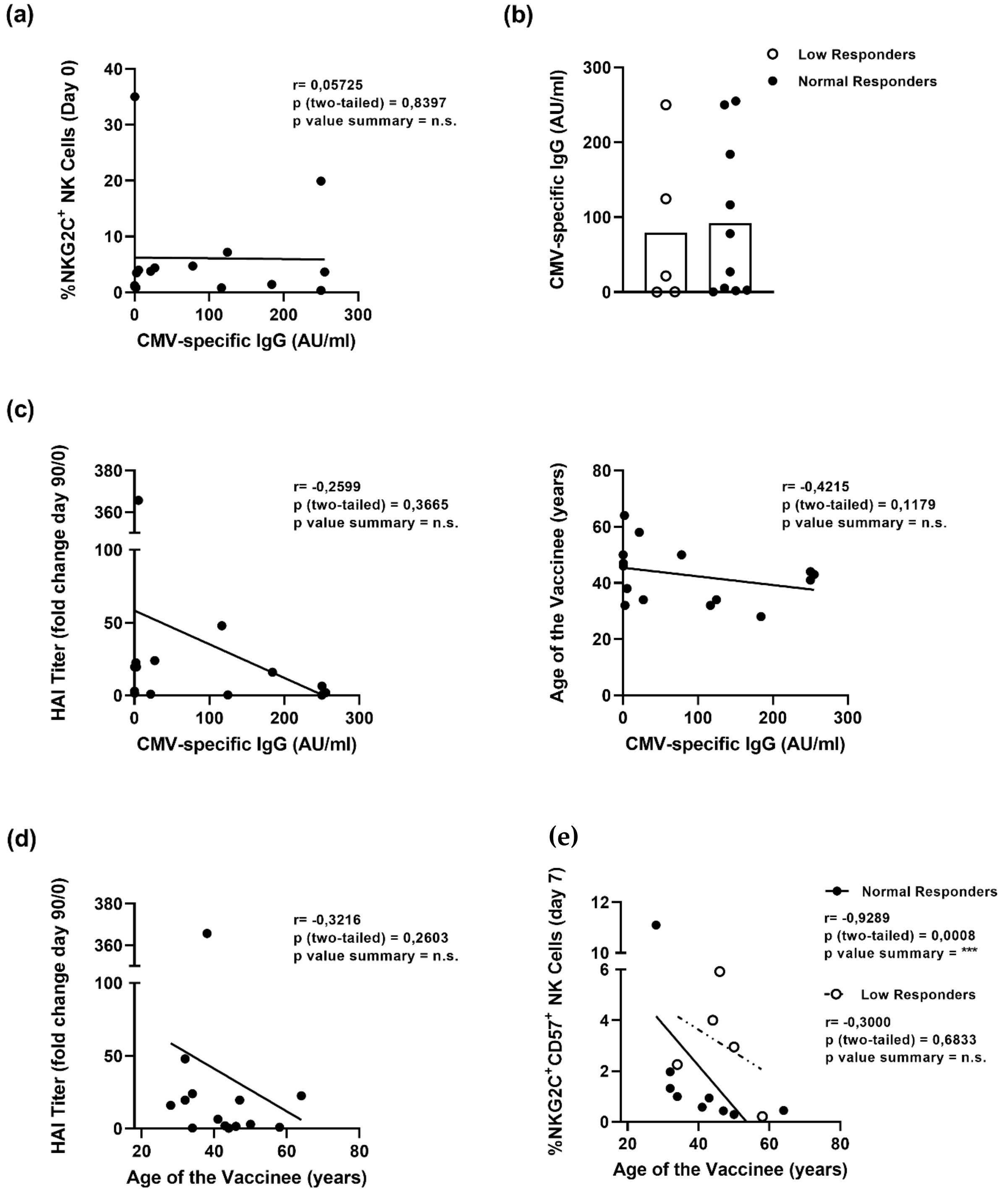

Figure S2. Correlation analyses. Serum samples were assessed for the titer (AU/ml) of CMV-specific IgG antibodies. (**a**) Correlation of NKG2C+ NK cells (percentage) before vaccination (day 0, assessed by flow cytometry) with the CMV-specific IgG titer. (**b**) CMV-specific IgG titer of vaccinees stratified into normal and low responders according to their HAI titer. (**c**) Correlation of the HAI titer fold change as well as the age of the vaccinees with the CMV-specific IgG titer. (**d**) Correlation of the HAI titer fold change and (**e**) of NKG2C+ NK cells (percentage) 7 days post vaccination (assessed by flow cytometry) with the age of the vaccinees. Asterisks denote significant values as calculated by Spearman correlation.

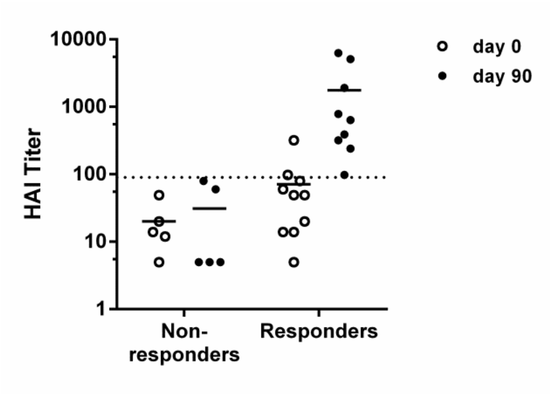

Figure S3. Classification of vaccine responders and non-responders. The vaccinated healthy volunteers were classified into responders and non-responders according to their HAI-titer 90 days after a single dose of the pandemic vaccine. The dashed line depicts the cut off-value dividing the two vaccine responsiveness groups.
